# Supplementary material for: MetaRibo-Seq measures translation in microbiomes
Source: Nat Commun. 2020 Jun 29;11:3268. doi: 10.1038/s41467-020-17081-z (PMC7324362; doi:10.1038/s41467-020-17081-z)
Supplement: Supplementary file 10 — Supplementary Data 7 [file 41467_2020_17081_MOESM10_ESM.zip › File2/Confidence_High_Taxonomy/144381_out.krona.html]

Javascript must be enabled to view this page.

members
magnitude
magnitudeUnassigned
count
unassigned
taxon
rank

144381\_out

2

2
superkingdom
2

1239
phylum
2

91061
class
2

2
order
186826

1300
family
2

genus
1301
2

1

SRS019028\_contig\_number\_contig-100\_2056.2056
1318
species


SRS062878\_contig\_number\_16591
1
species
28037
